# Lessons from Bacillus Calmette-Guérin: Harnessing Trained Immunity for Vaccine Development

**DOI:** 10.3390/cells9092109

**Published:** 2020-09-16

**Authors:** Samuel T. Pasco, Juan Anguita

**Affiliations:** Inflammation and Macrophage Plasticity Laboratory, CIC bioGUNE-Basque Research and Technology Alliance (BRTA), Parque Tecnológico de Bizkaia, 48160 Derio, Spain; spasco@cicbiogune.es

**Keywords:** Bacillus Calmette-Guérin (BCG), innate immune memory, mucosal immunity, vaccine

## Abstract

Vaccine design traditionally focuses on inducing adaptive immune responses against a sole target pathogen. Considering that many microbes evade innate immune mechanisms to initiate infection, and in light of the discovery of epigenetically mediated innate immune training, the paradigm of vaccine design has the potential to change. The Bacillus Calmette-Guérin (BCG) vaccine induces some level of protection against Mycobacterium tuberculosis (Mtb) while stimulating trained immunity that correlates with lower mortality and increased protection against unrelated pathogens. This review will explore BCG-induced trained immunity, including the required pathways to establish this phenotype. Additionally, potential methods to improve or expand BCG trained immunity effects through alternative vaccine delivery and formulation methods will be discussed. Finally, advances in new anti-Mtb vaccines, other antimicrobial uses for BCG, and “innate memory-based vaccines” will be examined.

## 1. Introduction

Vaccine strategies typically aim to generate and subsequently preserve an antigen-specific, B- and/or T-cell-mediated immune response against the targeted pathogens. However, studies focused on live attenuated vaccines like the Bacillus Calmette-Guérin (BCG), measles vaccine, oral polio vaccine (OPV), and smallpox vaccine have described beneficial nonspecific effects that induce reduction in the overall mortality associated with infection [1]. Nonspecific effects mediated by the adaptive immune system have been described and include: cross-reactive T-cell receptors and/or antibodies, potentiation of classical cell-mediated immunity through increased general cytokine signaling, and bystander activation of memory components through a specific cytokine milieu [2]. In contrast, the observed phenomena suggest mechanisms in adaptive-independent innate immune memory.

These enhanced responses, known as trained immunity, involve epigenetic rewiring of innate immune cells that result in long-term adaptation and facilitate amplified responses to stimuli [3]. Complex stimuli, like BCG, and simple pathogen-associated molecular patterns (PAMPs), like β-glucan, can both produce trained immunity effects that persist days and months after in vitro and in vivo administration, respectively. The discovery of trained immunity opens up the possibility of designing vaccines that, at least in part, stimulate and prime innate cells to enhance their response against the target and other pathogens. This review will explore the nonspecific effects of BCG and its effects on the innate immune system. Specifically, we will describe innate mechanisms of BCG-induced protection that produce trained immunity in monocytes and macrophages. We will also explore potential strategies for BCG to enhance innate memory responses through its formulation and delivery, especially to mucosal tissues. Finally, we will extrapolate beyond BCG to explore other vaccine strategies that target and induce trained immunity.

## 2. BCG Vaccination and Tuberculosis Infection

BCG is the only approved vaccine against Mycobacterium tuberculosis (Mtb). In use since the 1920′s and given to approximately 100 million infants annually, the live attenuated strain of M. bovis provides children with >50% and >80% protection against lung disease and disseminated tuberculosis (TB), respectively [4]. When BCG was first developed, oral administration was standard until 1927, as M. bovis naturally infects the gastrointestinal tract [5]. Currently, the BCG vaccine is administered as a solution of lyophilized bacilli reconstituted in saline, without any additives.

Mtb is the most prevalent infectious pathogen on the planet. According to the World Health Organization (WHO), 10 million people became sick and over 1.4 million people died from TB in 2018, while estimates predict that up to one third of the human population has a latent TB infection (LTBI) [6]. Natural infection with Mtb typically begins when microbes enter the lungs and encounter pulmonary phagocytic cells. Mtb survives intracellularly before T-cells arrive by exerting immunosuppressive effects that inhibit phagosome maturation, lysosome fusion, production of reactive oxygen species, major histocompatibility class II (MHC-II) antigen presentation, and apoptosis [7]. Localized control of Mtb infection results in the formation of complex, multicellular granulomas that contain the LTBI. The complex Mtb cell envelope also contributes to its ability to survive intracellularly, which contains a complex polysaccharide outermost capsule, an asymmetrical outer membrane, a peptidoglycan-arabinogalactan complex covalently bound to the outer membrane, and the inner membrane [8]. The complex and incompletely understood pathogenic mechanisms of Mtb complicate both vaccination and treatment.

BCG vaccination does not confer absolute protection against Mtb, and the lack of immune correlates of Mtb protection presents challenges for improved vaccine design. BCG and other parenteral TB vaccine strategies appear to induce a “near-natural immunity”: they imitate the naturally occurring infectious events after the initiation of Mtb infection, with vaccine-activated adaptive cells circulating systemically instead of residing in the lung parenchyma and without altering the lung environment [7]. Because there is no evidence demonstrating that BCG can prevent the infection from establishing, it is assumed that BCG helps prevent the progression to disease [4]. BCG does provide protection against the development of disease, as revaccination in a high-risk setting conferred protective effects, as demonstrated by a significantly reduced sustained rate of interferon-gamma (IFNγ) release assay (IGRA) conversion compared to the placebo [9]. Understanding the mechanisms of BCG-induced protection would accelerate TB vaccine development by elucidating the protective immune response to Mtb, which would illuminate pathways for new and revamped treatments [10].

Enhanced innate responses may protect against Mtb outside the context of vaccination. About a quarter of TB household contacts are early clearers, as demonstrated by testing consistently negative on IGRA [11]. Additionally, the IGRA of BCG-vaccinated contacts was less likely to convert than unvaccinated contacts, but the BCG-induced protection decreased as age and likelihood of exposure increased. In a mycobacterial growth inhibition assay (MGIA) using BCG, peripheral blood mononuclear cells (PBMCs) from individuals recently exposed to Mtb had an increased ability to control BCG growth compared to individuals with LTBI [12]. Elucidating these mechanisms and harnessing this capacity could improve BCG and future vaccines.

Although not completely protective against Mtb, BCG vaccination correlates with overall beneficial protective effects. BCG vaccination is associated with a decrease in all-cause mortality [13]. Children with neonatal BCG vaccination have lower mortality than unvaccinated children, regardless of Mtb-exposure status [14]. Low birth weight infant mortality from BCG-vaccinated neonates in the first month of life was reduced, albeit insignificantly, by 17% due to fewer incidences of infections [15]. Additionally, BCG revaccination for adolescents in a high-risk setting significantly reduced the overall incidence of upper respiratory tract infections [9], while BCG vaccination in elderly patients decreased the frequency of general infection that was most beneficial against respiratory infections of probable viral origin [16].

## 3. Nonspecific and Enhancing Effects of BCG-Induced Trained Immunity

**Figure 1:**
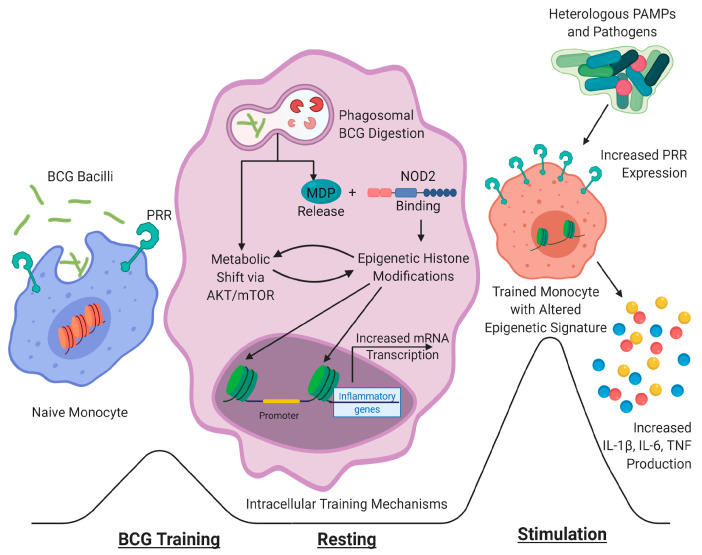
Mechanisms of in vitro Bacillus Calmette-Guérin (BCG) training. Naive monocytes are stimulated with BCG, then rested for several days. Phagosomal digestion of BCG causes the release of muramyl dipeptide (MDP), as well as a metabolic shift towards glycolysis through the Protein kinase B (AKT)/mammalian target of rapamycin (mTOR) pathway. MDP binds nucleotide-binding oligomerization domain-containing protein 2 (NOD2) to induce epigenetic histone alterations, which are interconnected with the metabolic changes. Epigenetic modifications result in increased access to the promoter regions of genes related to inflammatory pathways, such as cytokine and pattern recognition receptors (PRRs). Trained monocytes express higher levels of PRRs and produce increased levels of cytokines following stimulation with heterologous pathogens or pathogen-associated molecular patterns (PAMPs).

Trained innate immunity is characterized by enhanced cytokine production following in vitro stimulation with unrelated pathogens and non-specific stimuli. An experimental model established a protocol for human primary monocytes, where training stimuli are incubated with cells for 24 h, rested for up to seven days, and re-stimulated with unrelated stimuli (Figure 1) [17]. PBMCs trained with live BCG produced high levels of interleukin (IL)-6 and tumor necrosis factor (TNF), and increased reactive oxygen species (ROS) production and metabolic shifts dependent on training and resting time. Inactivated BCG induces trained immunity, though at a lower magnitude than live BCG [18]. Increased IL-6 production following in vitro BCG training and lipopolysaccharide (LPS) stimulation occurs in both neonate and adult monocytes [19].

Innate immune cells from BCG-vaccinated subjects have enhanced responses upon re-stimulation, particularly with PAMPs and heterologous pathogens. However, these effects do not appear immediately, as whole blood from newborns vaccinated within a week of birth contained higher IL-6 concentrations and produced fewer cytokines and chemokines following stimulation with different Toll-like receptor (TLR) agonists and heterologous pathogens than unvaccinated blood [20]. Trained immunity effects likely take more than a week to take effect, as stimulation with TLR agonists of whole blood from low birth weight infants four months post vaccination did increase production of IL-1β, IL-6, TNF, and IFNγ [21]. Similarly, at the same time point, healthy infants’ whole blood stimulated with heat killed heterologous pathogens, and other PAMPs demonstrated increased production of 11 cytokines and chemokines and suppressed production of six cytokines, with distinct responses to each stimulus [22]. mRNA transcription and secretion of IL-1β and TNF from PBMCs of vaccinated adult volunteers increased following heterologous stimulation, up to three months post vaccination [23]. PBMCs from volunteers vaccinated with gamma-irradiated BCG (γBCG) only demonstrated increased cytokine production in response to Mtb antigens, with no significant trained immunity effects observed after two weeks and three months [18]. BCG vaccination in adults over 50 induced trained immunity, with larger effects seen in those with a positive IGRA at baseline [24]. Increased TNF and IL-6 secretion following pathogen stimulus returned to baseline after one year, except for LPS-induced TNF and IL-1β [25]. Additionally, monocyte-derived macrophages isolated three weeks post BCG vaccination from a subset of subjects, deemed “responders”, demonstrated enhanced containment of virulent Mtb replication [26]. Furthermore, stimulation of PBMCs from both vaccinated and unvaccinated early clearers produced more cytokines following heterologous bacterial stimulation [27].

BCG immunization in humans outside the context of preventing Mtb has demonstrated altered immune responses when administered with other pathogens, in both vaccination and challenge experiments. BCG inoculation to naive volunteers before vaccination with attenuated yellow fever virus (YFV) resulted in lower viremia, without affecting anti-YFV humoral responses, demonstrating that its effects only modulate the anti-YFV innate response [28]. Interestingly, while PBMCs from vaccinated individuals released more cytokines following ex vivo stimulation, unvaccinated individuals had higher systemic cytokine levels. BCG can improve adaptive responses, as BCG administration before vaccination against the 2009 H1N1 pandemic influenza strain improved antibody responses against the virus [29]. BCG-naive volunteers challenged with Plasmodium falciparum developed overall higher parasitemia and earlier symptoms, though a subgroup had earlier monocyte activation and lower parasitemia [30]. However, in an experimental endotoxemia immunoparalysis trial, γBCG did not confer protection in vivo nor altered leukocyte responses ex vivo [31].

## 4. Mechanisms of BCG-Induced Protective and Trained Immunity

**Figure 2:**
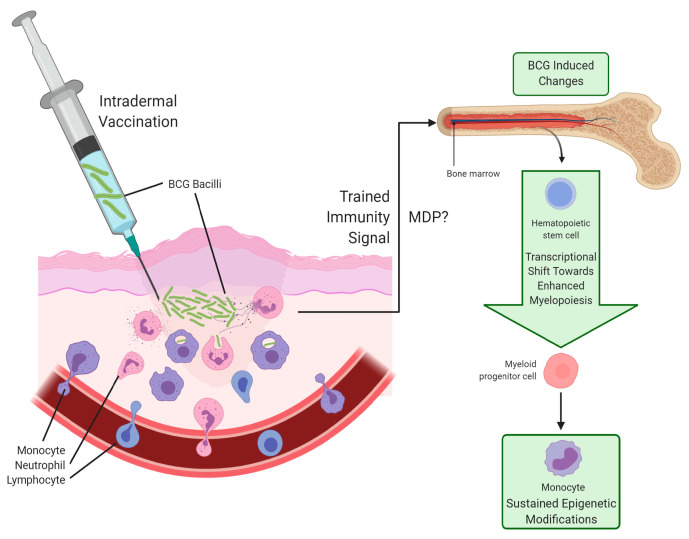
Intradermal BCG vaccination and establishment of trained immunity in vivo. Lyophilized BCG bacilli reconstituted in saline is administered by intradermal injection. Neutrophils, monocytes, and lymphocytes are the predominant cells that infiltrate the vaccination site, where live BCG can persist for up to four weeks. A yet uncharacterized signal (potentially MDP) causes a change in the hematopoietic stem cells of the patient’s bone marrow, which induces a transcriptional shift resulting in increased myelopoiesis. As early as two weeks following inoculation, monocytes have a trained immunity phenotype with sustained epigenetic changes that last up to a year.

Following standard intradermal inoculation, resident epidermal macrophages are the first immune cells to encounter BCG. Before recruiting other immune cells, these macrophages phagocytose the bacilli and bind mycobacterial PAMPs through pattern recognition receptors (PRRs), including complement receptor 3, TLR2, and TLR4 [8,32]. One week post vaccination, blister cell infiltrates demonstrated high frequencies of neutrophils, monocytes, and lymphocytes with low frequencies of dendritic and natural killer cells (Figure 2) [33]. Furthermore, live BCG can persist at the inoculation site up to four weeks post vaccination. Seven days after vaccination in a mouse model, the lungs contained higher percentages of extravasated CD11b^+^F4/80^+^ monocytes and CD11b^+^CD14^+^ cells, demonstrating an increased load of phagocytic cells [34]. Interestingly, in humans, vaccination reduces expression of CD11b and Human Leukocyte Antigen, DR isotype (HLA-DR) in CD206^+^/CD169^+^ alveolar macrophages both after two weeks and three months [35]. Unfortunately, trained immunity effects could not be observed in these macrophages, due to activation following sputum collection.

While mycobacterial PAMPs bind to many host PRRs, the induction of BCG-induced trained immunity depends on the key cytosolic PRR nucleotide-binding oligomerization domain-containing protein 2 (NOD2). Stimulation of macrophages deficient in NOD2 with BCG did not result in increased cytokine production following heterologous stimulation, demonstrating the critical importance of this signaling pathway in establishing trained immunity [23]. Similarly, γBCG stimulation of monocytes from patients with a different homozygous NOD2 mutation also resulted in a decreased induction of trained immunity [18]. NOD2 binds muramyl dipeptide (MDP), the minimal structural component of peptidoglycan necessary for biological action, which is released following lysosomal digestion of bacteria or intracellular bacterial growth [36]. While BCG vaccination causes an increase in circulating MDP concentration, the baseline MDP concentration strongly and positively correlates with IL-1β, IL-6, and TNF production following nonspecific stimulation of PBMCs three months post vaccination [37]. Additionally, MDP concentration did not affect the specific IFNγ-mediated antimycobacterial response, demonstrating that the adaptive response relies on separate mechanisms than trained immunity. Interestingly, BCG vaccination in NOD-deficient mice seven days prior to challenge, regardless of inoculation route, induced similar immunity to wild-type mice, as measured by mycobacterial loads [34], thus showing that its role primarily pertains to initiating memory.

BCG vaccination causes changes to the cellular metabolic pathways, in both in vitro training assays and in vaccinated subjects. In vitro, BCG training induces elevated glucose consumption, lactate release, oxygen consumption rates, and glutamine metabolite concentrations, which result from increased phosphorylation of AKT, mammalian target of rapamycin (mTOR), and other downstream effector proteins [38]. Additionally, inhibition of glycolytic metabolism, glutamine metabolism, and the downstream signaling molecules prevented the training effects. Furthermore, ex vivo PBMC stimulation from BCG-vaccinated subjects and unvaccinated subjects treated with metformin, an mTOR inhibitor, demonstrated increased lactate concentrations in the supernatants and inhibited BCG-induced trained immunity respectively, thus confirming in vivo the in vitro observations.

BCG-induced training causes epigenetic histone modifications that remodel chromatin, and the resulting transformational changes allow differential gene expression for a more robust response. In vitro, BCG training induced trimethylation increase at histone H3 lysine 4 (H3K4), an activator marker, and decrease at histone H3 lysine 9 (H3K9), a repressor marker, of promoters for TNF, IL-6, mTOR, and enzymes for glycolysis and glutaminolysis [38]. Additionally, mRNA expression from mTOR and metabolic enzymes subsequently increased, but chemical inhibition of these proteins abrogated training effects, demonstrating the interconnectivity between metabolic and epigenetic changes. γBCG-trained PBMCs also upregulated the H3K4 position at TNF and IL-6 promoters, though not as robustly as live BCG [18]. In vitro training in the presence of all trans-retinoic acid, which increases expression of the inhibitory histone methyltransferase SUV39H2 responsible for H3K9 trimethylation, demonstrated dose-dependent repression of cytokine promoter regions [39]. Inhibiting histone methyltransferase activity, but not demethylase activity, during in vitro BCG training prevented enhanced cytokine responses following nonspecific stimulation [23].

BCG responders had distinct methylation patterns at three weeks, and four and eight months post vaccination, which were tied to immune pathways such as “innate immune response” and “leukocyte activation” [26]. Vaccination increased the accessibility of several genes associated with inflammatory processes while decreasing accessibility of genes related to lymphoid development and anti-inflammatory processes [40]. PBMCs from vaccinated subjects demonstrated increased H3K4 trimethylation at promoters for IL-6, TNF, and TLR4 [23]. Vaccination also induces acetylation at histone H3 lysine 27 promoters and regulators for inflammatory, cytokine, G protein-coupled receptor, and protein kinase genes [28].

In addition to enhanced cytokine responses to heterologous stimulation, circulating monocytes express higher levels of PRRs. PBMCs from vaccinated subjects express increased levels of TLR4 and CD11b three months post BCG [23]. CD11 and CD14 were persistently increased throughout a year follow-up, while TLR4 and mannose receptor expression increased one year post vaccination [25].

Effects of trained immunity can last up to a year, even though monocytes have a 5–7 day half-life [3], indicating that changes could be made in progenitor cells. Intravenously delivered BCG can sustainably reprogram murine hematopoietic stem and progenitor cells (HSPCs) in the bone marrow (BM) to enhance myelopoiesis, and the resulting epigenetically modified macrophages and monocytes provided protection against Mtb in vitro and in vivo, respectively [41]. A groundbreaking study demonstrated similar changes in human BM following BCG vaccination in healthy naive subjects, where the upregulated transcriptional shift towards myelopoiesis and subsequent increased cytokine production from PBMCs following nonspecific stimulation was confirmed [40]. Specifically, the epigenetic modifications in HSPCs guarantee continued modification for circulating monocytes 90 days after vaccination (Figure 2). Furthermore, the identification of the hepatic nuclear factor family of transcription factors as master regulators of trained immunity induction in HSPCs provides mechanistic insight of the process. It remains to be seen by what mechanism these BM changes occur and for how long these changes last, though MDP likely plays a role in establishing trained immunity in these cells.

## 5. Optimizing BCG Formulation and Delivery to Augment Innate Responses

While BCG has been used for almost 100 years, it does not confer complete protection against Mtb. New strategies should be explored to address the many factors that could enhance the uniformity and effects of BCG. For example, more than 14 different strains of BCG exist, but considerable variability in mycobacterial viability, RNA content, and activation of cytokine responses exist, likely contributing to the vaccine’s inconsistent effects [42]. Additionally, intradermal administration of BCG has well-documented limitations in its ability to protect against disease. One factor that could contribute to these limited effects, particularly in areas of low vaccine efficacy, is exposure to non-Mtb environmental mycobacteria. Intradermally vaccinated mice chronically exposed to oral M. avium produced more T regulatory cells and immunosuppressive IL-10 while decreasing IFNγ production [43]. Therefore, exploring different inoculation routes and vaccine composition could provide improvements to the protective effects.

### 5.1. Vaccine Delivery

Intravenous delivery of BCG has been explored. Potential benefits include more direct vaccine delivery to the pulmonary tissues where natural infection begins as well as to the BM where HPSCs reside. Intravenous BCG delivery to nonhuman primates (NHPs) resulted in increased IFNγ production and CD4^+^ T-cell frequencies while reducing pathology and improving survival compared to other vaccination methods [44]. NHPs demonstrated thorough protection against Mtb challenge following intravenous BCG administration [45]. Specifically, intravenous delivery resulted in major increases in antigen-responsive adaptive cells in the bronchoalveolar lavage (BAL), lung lymph nodes, lung parenchymal tissues, blood, and spleen. However, there was no evidence of trained immunity, as PBMCs from both intradermally and intravenously vaccinated NHPs stimulated with non-Mtb antigens failed to produce increased levels of TNF, IL-1β, or IL-6. This represents puzzling results inconsistent with murine and intradermal human studies. However, as of this publication, no studies have explicitly established BCG-induced trained immunity effects in NHPs.

Mucosal immunization with BCG has been investigated. The total mucosal surface area is about 200 times larger than skin, and vaccination at the sites of pathogen invasion could generate a protective immunological response [46]. While oral BCG was the initial delivery method almost a century ago, oral vaccines generally must survive the acidic environment of the stomach and run the risk of generating tolerance without an adjuvant [47]. That said, comparing intradermal and oral delivery in humans, oral BCG induced stronger mucosal responses, as measured by Mtb-specific bronchoalveolar lavage (BAL) T-cells and secretory Immunoglobulin A (IgA), though intradermal BCG resulted in stronger systemic Th1 responses [48]. These results also demonstrate that mucosal vaccination at one site can produce a response at a distal mucosal surface. 

Respiratory delivery of BCG would result in mucosal vaccination at the infection site, allowing tissue-resident immune cells more direct access to the vaccine antigens. Imprinting protective effects on lung innate cells to respond better to an Mtb encounter could help phagocytic cells resist Mtb-directed immunosuppression [7]. Studies have explored airway delivery of BCG (such as through aerosol, intratracheal, or pulmonary vaccination). Aerosol BCG vaccination in young calves induces a trained immunity phenotype in circulating PBMCs, as demonstrated by increased cytokine production after PAMP stimulation [49]. However, this phenotype did not appear in alveolar macrophages, potentially due to the immunosuppressive nature of these cells. In NHPs, pulmonary vaccination followed by repeated Mtb exposure reduced lung pathology [50]. NHPs that received an intratracheal BCG boost after intradermal BCG vaccination also reduced pulmonary disease [44]. Aerosol BCG vaccination in mice conferred protection against Mtb challenge through increased IFNγ levels and T-cell recruitment into the lung, even in the presence of environmental mycobacteria [43].

Furthermore, trained immunity is affected by the timing of BCG administration. In clinical trials in Guinea-Bissau, BCG immunization to low-weight infants administered between November and January, during peak malaria infections, both beneficially reduced all-cause neonatal mortality and resulted in stronger responses to heterologous stimulation in whole blood assays, suggesting that there may be seasonal considerations for BCG immunization [51]. Trained immunity effects, as well as specific adaptive responses, were stronger when BCG was administered to adult volunteers in the early morning versus later in the morning, while evening vaccination produced almost no enhancement in specific and nonspecific effects [52].

The efficacy of BCG and other vaccines may be affected by the administration schedule, as simultaneous BCG and oral polio vaccine (OPV) vaccination in infants reduced in vitro cytokine responses at 6 weeks and in vivo responses to Mtb-purified protein derivative at two months [53]. Tetanus-diphtheria-pertussis-inactivated polio vaccine-induced immunosuppression in adult volunteers was rescued by BCG administration concurrently or after three months [54].

### 5.2. Vaccine Formulation

Chemical alteration of the BCG vaccine could enhance innate responses and help establish trained immunity, through selective nutrient culturing or chemical treatment. BCG cultured in a phosphate-deficient media resulted in increased expression of glycoprotein adhesins that facilitated macrophage phagocytosis [55]. Alveolar lining fluid (ALF), which Mtb passes through upon infection, alters BCG immunogenicity, as mice subcutaneously vaccinated with ALF-treated BCG demonstrated reduced Mtb burden and lung inflammation [56]. BCG itself may be too pathogenic for pulmonary inoculation, but removing inflammatory lipids by petroleum ether treatment prior to murine aerosol vaccination demonstrated improved protection against infection and reduced inflammation in the lung [57]. Interestingly, in vitro macrophage stimulation with the delipidated BCG resulted in reduced mycobacterial uptake, intracellular growth, and cytokine production compared to standard BCG. 

These effects could be further enhanced if bacilli were delivered with phagocytosis-promoting compounds or adjuvants. Chitosan, the second most abundant natural biopolymer found in some microbial cell walls and exoskeletons of crustaceans and insects, can accumulate in and activate phagocytic cells such as macrophages [58]. Novel BCG-loaded chitosan vaccine formulations doubled cellular uptake in vitro and, when delivered intranasally, increased murine Th1 responses compared to subcutaneous inoculation [59]. Many approved vaccines in clinical use contain adjuvants to help stimulate and guide the protective immune response. Chitosan-based nanoparticles containing TLR3 agonist poly(I:C) administered with BCG to murine BMDMs in vitro synergistically increased the BCG-induced responses 60-fold towards a pro-inflammatory phenotype, including an increase in cytokine and nitric oxide (NO) production [60]. Moreover, due to its critical importance in establishing trained immunity effects, the addition of MDP or other NOD2 agonists as an adjuvant to BCG’s formulation could potentially strengthen or even guarantee the inception of nonspecific effects.

## 6. Harnessing and Improving BCG-Elicited Trained Immunity against Mtb and Beyond

Enhancing and utilizing BCG’s trained immunity effects in future vaccines against Mtb should be a priority and utilizing recombinant technologies to enhance BCG immunogenicity or reduce Mtb pathogenicity presents a unique opportunity to enhance anti-Mtb candidates. A recombinant BCG with overexpression of Mtb di-adenylate cyclase, which produces bacterial secondary messenger cyclic di-AMP, demonstrated comparable protection to BCG-immunized mice, while cellular analyses demonstrated increased IL-6 production following Mtb challenge and higher expression of H3K4 trimethylation than BCG [61]. Live-attenuated *M. tuberculosis* Vaccine Candidate (MTBVAC), the first genetically modified, live attenuated vaccine based on Mtb which has demonstrated safety and efficacy in initial clinical trials, induces trained immunity effects in vitro through shifts in metabolism and epigenetic changes at proinflammatory promoters, and can protect subcutaneously vaccinated mice from lethal intranasal doses of Streptococcus pneumoniae [62]. Furthermore, vaccination with RUTI, a liposomal formulation containing cellular fragments of Mtb bacilli cultured to mimic an intra-granulomatous latency environment that has demonstrated poly-antigenic responses in clinical trials of patients with LTBI, caused a shift in murine monocyte phenotype associated with enhanced mycobacterial growth inhibition assay (MGIA) responses [63].

Mobilizing trained immunity effects could improve other vaccines by using BCG as a primer, adjuvant, or vector. Murine rectal administration of BCG prior to subcutaneous vaccination with autoclaved Leishmania major, an intra-macrophage parasite, resulted in higher NO production associated with peritoneal macrophage NO synthase induction, both four and eight weeks after challenge infection [64]. Administration of both BCG and hepatitis B (HBV) vaccine to young mice enhanced anti-HBV antibody titers [65]. Interestingly, this study also provided evidence of synergistic IL-1β production following in vitro BCG and HBV stimulation of human preterm, term, and adult whole blood. While recombinant BCG vaccines have been explored with promising results for viral, bacterial, and parasitic pathogens, it is unclear whether the cross-protective effects of the wild-type are present, with no evidence of heterologous effects against distinct pathogens [66].

Harnessing BCG for nonspecific protection against viral infections, particularly during pandemics, could confer protection until appropriate therapeutic interventions become available. Due to its beneficial nonspecific effects, it has recently been proposed to use BCG to protect against novel SARS-CoV-2 infection while a specific vaccine is being developed [3]. As of this publication, at least seven clinical trials are active or recruiting subjects for placebo-controlled studies in healthcare workers or the elderly (clinicaltrials.gov: NCT04327206, NCT04328441, NCT04348370, NCT04379336, NCT04414267, NCT04417335, NCT04475302). It is not yet clear how trained immunity could affect SARS-CoV-2 infection, but in responses to a digital survey from a cohort of individuals vaccinated with BCG within the past five years, significantly fewer vaccinated subjects self-reported sickness than control subjects [67]. Additionally, an epidemiological analysis of European countries demonstrated a powerful significant correlation between BCG index (a quantifiable estimate of universal BCG administration) and COVID-19 mortality, whereby every 10% increase in BCG index associated with a 10.4% decrease in mortality [68].

BCG has demonstrated beneficial effects against influenza, another virus with pandemic potential. BCG immunization in immunized mice conferred significant protection against intranasal influenza challenge, with intranasal vaccination stronger than the intraperitoneal route [69]. Although intravenous murine BCG delivery prompted trained immunity effects but did not significantly protect against experimental H7N9 influenza [70], pulmonary aerosol BCG delivery before lethal H1N1 influenza challenge completely protected mice by increasing the capacity of alveolar phagocytes to clear apoptotic cells, thus protecting from influenza-induced pneumonia [71].

## 7. Future Directions in Exploiting Trained Immunity

**Figure 3:**
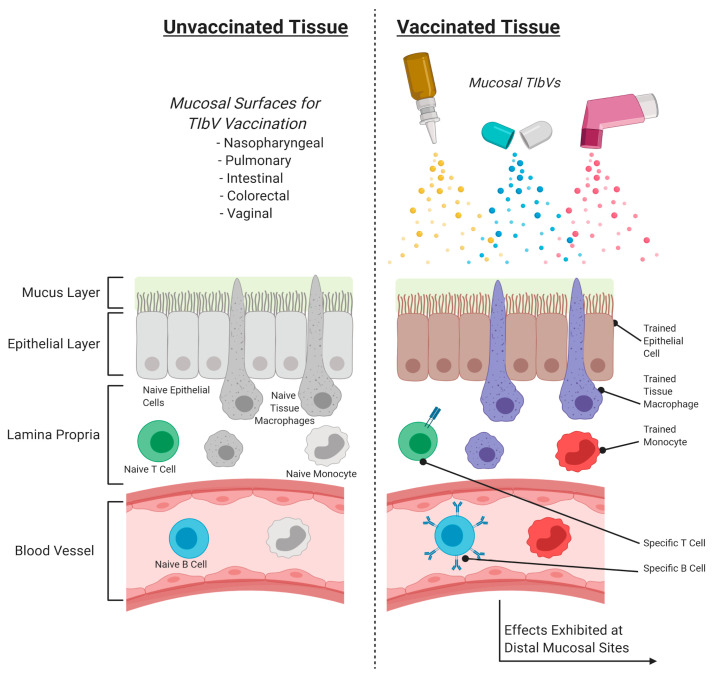
Proposed mechanism of action for hypothetical mucosal trained immunity-based vaccine (TIbV). TIbV vaccination at mucosal surfaces could induce trained immunity to tissue-resident and peripheral innate cells and epithelial cells, while producing a complementary adaptive response. Vaccination at one mucosal site does demonstrate effects at distal mucosal sites.

Designing vaccines and other future therapeutics that intentionally harness innate nonspecific effects would be a promising strategy to not only improve current treatments, but also to create new options to address increasingly resistant pathogens. BCG fails by relying on adaptive responses that play little to no role in the preliminary steps of Mtb infection; but, by focusing on innate responses, better vaccines could be designed that offer better protection. Such treatments could be considered “trained immunity-based vaccines” (TIbV), anti-infectious vaccines containing trained immunity inducers and pathogen antigens effective versus the target and heterologous pathogens [72]. A respiratory or other mucosal TIbV could produce a sterilizing immunity that prevents the development of an active infection or the establishment of latent colonization (Figure 3) [7]. These responses would predictably still generate an adaptive response which would complement or even enhance innate responses.

Sui and colleagues [73] developed a mucosal human immunodeficiency virus vaccine that fits many of the TIbV criteria, which was delivered intracolorectally to NHPs and contains both a modified vaccinia Ankara-simian immunodeficiency virus and a peptide vaccine, with IL-15, TLR2/6, TLR3, and TLR9 agonists as adjuvants. While the vaccine did confer significant protection against simian-human immunodeficiency virus (SHIV) intrarectal challenge, humoral and T-cell responses alone did not correlate with protection. Instead, along with a vaccine-induced alteration in gut microbiome, an influx of myeloid cells to colorectal mucosa, which produced increased TNF and IL-6 upon ex vivo stimulation with SHIV, correlated with protection [73]. These results demonstrate that mucosal trained immunity can be induced by a vaccine and can confer protection. However, this study did not explore nonspecific effects of the mucosal monocytes, which could solidify this vaccine’s status as a TIbV.

Designing mucosal TIbVs should focus on a “whole-of-mucosa” approach that considers the immunomodulatory properties of non-immune cellular components, as many mechanisms could be exploited for enhanced vaccine performance. Epithelial cells can develop trained immunity, as demonstrated when primary epithelial cells (PECs) treated with Pseudomonas aeruginosa flagellin increased inflammatory responses to live, unrelated stimuli as a result of epigenetic modifications [74]. PECs stimulated by Mtb-infected monocytes or alveolar macrophages express antimycobacterial peptides and defensins and promote neutrophil influx [75]. BCG-stimulated PECs increased CXCL8 production and neutrophil influx, with increased IL-6 production when proinflammatory cytokines IFNγ and IL-17A were administered with BCG [76]. Additionally, Mtb can adapt to infection in alveolar epithelial cells, where they undergo phenotypic transformation to become more invasive and replicative, and therefore could be targeted [77]. The immunoprotective capacity of non-immune mucosal components demonstrates their importance and should be taken into consideration when designing mucosal vaccines.

Some clinically available vaccines and other immunomodulatory therapies containing lysates of polybacterial formulations could be considered mucosal TIbVs, as they modulate and maintain innate immune responses and confer protection against nonspecific pathogens at mucosal sites, such as the respiratory and urogenital tracts [72]. 90% of patients taking three months of sublingual bacterial preparation MV140, consisting of four common inactivated uropathogens for recurrent urinary tract infections, were protected from relapse infections, while every patient prescribed six months of prophylactic antibiotics experienced relapses [78]. Patients with chronic, recurrent respiratory infections have lower surface expression levels of TLR2 and CD14 on their circulating monocytes, but oral administration of Respivax, a formulation of six respiratory pathogens, restores these levels to match the healthy controls [79], demonstrating trained immunity-like effects. In vitro stimulation with polyvalent bacterial lysate, prepared with six common respiratory pathogens, induced dose-dependent production of NO in murine alveolar macrophages and increased transcription of pro-inflammatory chemokines and cytokines, NO synthase, and antimicrobial peptides in human epithelial cells [80]. Patients with chronic bronchitis have reduced alveolar macrophage activity regardless of smoking history, and treatment with Broncho-Vaxom (OM-85), an oral capsule containing eight strains of bacterial extracts, significantly increased macrophage activity in the BAL, due to stimulation by IFNγ [81,82]. In vitro stimulation with the OM-85 trained murine macrophages for intracellular killing of parasitic Leishmania enriettii [83]. Orally administered OM-85 protected mice from aerosol H1N1 influenza and intraperitoneal Salmonella typhimurium infections.

## 8. Conclusions

In conclusion, the BCG vaccine’s demonstrated ability to establish trained immunity presents the opportunity to develop other vaccines that elicit similar responses. Research on the critical intracellular mechanisms of BCG-induced innate memory will help guide future anti-Mtb therapeutics to harness these beneficial nonspecific effects. Optimizing BCG should focus on vaccine formulation and delivery, particularly to mucosal sites, both of which could profoundly improve protection against Mtb and potentially other pathogens. In order to harness trained immunity effects for future vaccine candidates, researchers should consider designing mucosal TIbVs that prime both immune and non-immune cellular components for prophylactic vaccination and therapeutic treatment. This approach represents an avenue to address challenging bacterial infections beyond Mtb as a new strategy against antimicrobial resistance and challenging emerging infections.
